# Mohs micrographic surgery for basal cell carcinoma in Singapore: A retrospective review

**DOI:** 10.1016/j.jdin.2024.07.016

**Published:** 2024-08-23

**Authors:** Angelyn Chen Yin Lua, Dawn Ai Qun Oh, Hui Xin See Tow, Ren Hong Por, Hong Yi Koh, Choon Chiat Oh

**Affiliations:** a Department of Dermatology, Singapore General Hospital, Singapore; b Yong Loo Lin School of Medicine, National University of Singapore, Singapore, Singapore; c Duke NUS Medical School, Singapore, Singapore

**Keywords:** Asia, barriers, basal cell carcinoma, complications, cost-effectiveness, Mohs Micrographic Surgery, surgery

*To the Editor:* Basal cell carcinoma (BCC) has become increasingly common in Singapore over the years.^1^ Mohs micrographic surgery (MMS) is widely accepted as the treatment with the highest cure rate for BCCs.^2^ There is a paucity of data in Asia, with use of MMS reported in less than 20% of Asian countries. We present our experience with MMS for BCCs in a Singapore tertiary center.

We conducted a retrospective review of patients who underwent MMS for BCC in Singapore General Hospital from November 2019 to March 2024. Baseline demographics, tumor characteristics, and treatment details were collected. Outcomes included need for multiple-stage MMS, referral to other specialties for closure, and complications.

Sixty-four patients with a mean age of 73.8 years underwent MMS for BCC during the study period. Thirty-one (48.4%) were male and 33 (51.6%) were female. Four patients had MMS for 2 tumors in the same setting, and 3 had recurrent tumors.

All patients had BCCs located on the head or neck. Fifty-one (79.7%) patients had high-risk BCCs located on the central face, temple, nose, eyelids, chin, or ears. The most common BCC subtype was nodular (*n* = 51, 79.7%), followed by infiltrative BCC (*n* = 8, 12.5%).

Of patients, 73.4% (*n* = 47) required single-stage MMS, while 17 patients required 2 or 3 stages. Of multiple-stage MMS, 94.1% (*n* = 16) involved high-risk BCCs, and all were either nodular (*n* = 15) or infiltrative (*n* = 2) subtypes. Two out of three patients who had 3-stage MMS had BCCs located on or under the nose.

Defect closure was performed by the Mohs surgeon for most patients (*n* = 52, 81.3%). Plastic surgery or oculoplastics referrals were made for defect closure for 12 patients, 10 of whom had BCC either on the nose (*n* = 7) or near the eye (*n* = 3). Five patients requiring closure by other specialties underwent more than 1 stage for MMS. Three out of eight patients with infiltrative BCC required closure by other specialties.

Primary closure was employed for most defect repairs (*n* = 30, 46.9%). Other methods used are described in Table I.

**Table I:** Patient, tumor, and surgery characteristics

Age, y	
Mean (SD)	73.8 (11.4)
Minimum	34
Maximum	96
Sex, *n* (%)	
Male	31 (48.4%)
Female	33 (51.6%)
Immunocompromised, *n* (%)	
Yes	4 (6.3%)
No	60 (93.8%)
Antithrombotic, *n* (%)	
No antithrombotic	46 (71.9%)
Aspirin monotherapy	11 (17.2%)
Clopidogrel monotherapy	4 (6.3%)
Apixaban monotherapy	2 (3.1%)
Rivaroxaban monotherapy	1 (1.6%)
Tumor location, *n* (%)	
Area H	51 (79.7%)
Area M	13 (20.3%)
Tumor size, cm^2^	
Mean (SD)	0.9 (1.3)
Minimum	0.04
Maximum	6
Recurrent tumor, *n* (%)	
Yes	3 (4.7%)
No	61 (95.3%)
Histology proven pre-MMS, *n* (%)	
Yes	32 (50%)
No (dermoscopy proven)	32 (50%)
Histology on Mohs processing, *n* (%)	
Nodular	51 (79.7%)
Infiltrative	8 (12.5%)
Nodulocystic	1 (1.6%)
Basosquamous	1 (1.6%)
Number of stages, *n* (%)	
Mean	1.3
1	47 (73.4%)
2	14 (21.9%)
3	3 (4.7%)
Duration of surgery (start to close), min	
Mean	164.3
Defect size, cm^2^	
Mean (SD)	2.3 (2.4)
Minimum	0.2
Maximum	12
Defect repair type, *n* (%)	
Primary closure	30 (46.9%)
Secondary intention	4 (6.3%)
Skin graft	9 (14.1%)
Flap	27 (42.2%)
Defect closure specialty	
Dermatology (Mohs surgeon)	52 (81.3%)
Plastic surgery	11 (17.2%)
Oculoplastic surgery	1 (1.6%)
Complications	
Bleeding	3 (4.7%)
Wound dehiscence	1 (1.6%)
Wound infection	2 (3.1%)
Skin graft failure	1 (1.6%)

*MMS*, Mohs micrographic surgery.

Complications occurred in 6 (9.4%) patients. Three experienced bleeding, of whom one was on clopidogrel, while 2 were immunocompromised. Other complications were wound dehiscence (*n* = 1, 1.6%), minor wound infection (*n* = 2, 3.1%), and skin graft failure (*n* = 1, 1.6%). None of the patients with wound dehiscence or infection were immunocompromised.

Frozen section reading by the Mohs surgeon was highly concordant with subsequent histopathologist reading, with 2 (3.1%) nonconcordant readings.

In conclusion, our study found that MMS is highly effective for surgical management of BCCs in the Asian setting. The main complication experienced was bleeding in higher-risk patients on antiplatelets or immunosuppression.

However, MMS is not available in most Asian institutions. Table II describes possible challenges preventing MMS uptake and proposed solutions.

**Table II:** Challenges/barriers to MMS in Asia and proposed solutions

Challenges/barriers to MMS implementation in Asia	Proposed strategies to overcome challenges
Low volume of NMSCs in the region leading to lower need for dedicated MMS centers	Synergism: adding techniques from MMS, such as horizontal cuts during tissue processing to the intraoperative histological evaluation procedure instead of introducing a new MMS system entirely.^3^
	Improvement: non-MMS surgeons can adopt the above technique in treating NMSC at most health care institutions.^3^
Longer turnaround time and perceived low cost-effectiveness	Improved training: dermatologists can be certified and trained for both surgery and histopathological interpretation, reducing MMS duration and improving cost efficiency.^4^
	Division of labor within specific roles in the multidisciplinary team to improve cost-efficiency:^5^ • Mohs surgeon: excision, mapping, and reviewing of specimen • Secondary surgeon: specimen mapping, orientation, and handling
	Dermatopathologist: reviewing slides for surgical decisions • Optimizing treatments: patients with biopsy-proven skin cancers can be referred through a fast-track system for efficient resource utilization.^5^ • Optimizing resources: operating rooms can be made available to be used by other surgeons during specimen processing.^5^ • Prioritization: placing the emphasis on detection and removal of cancer lesions rather than maximizing esthetic preservation of skin tissue reduces MMS duration.^3^
Need for digital pathology services, which are more resource intensive and technologically challenging	Facilitation: utilizing improved surgical techniques or instruments to aid efficient processing of histological specimens.^3^
	Effective scheduling: ensuring the pathology department is available to interpret slides immediately upon receiving a Mohs map during MMS.^4^

*MMS*, Mohs micrographic surgery; *NMSC*, non melanoma skin cancer.

Limiting factors for our study are its retrospective nature and small case numbers due to cessation of MMS during the COVID-19 pandemic. Further longitudinal studies are warranted to examine the cost-effectiveness and identify other barriers to adoption of MMS in Asia.

## Conflicts of interest

None disclosed.
